# Novel Artificial Scaffold for Bone Marrow Regeneration: Honeycomb Tricalcium Phosphate

**DOI:** 10.3390/ma16041393

**Published:** 2023-02-07

**Authors:** Yasunori Inada, Kiyofumi Takabatake, Hidetsugu Tsujigiwa, Keisuke Nakano, Qiusheng Shan, Tianyan Piao, Anqi Chang, Hotaka Kawai, Hitoshi Nagatsuka

**Affiliations:** 1 Department of Oral Pathology and Medicine, Graduate School of Medicine, Dentistry and Pharmaceutical Sciences, Okayama University, Okayama 700-8525, Japan; 2 Department of Life Science, Faculty of Science, Okayama University of Science, Okayama 700-0005, Japan

**Keywords:** honeycomb TCP, bone marrow formation, scaffold, hematopoietic stem cell (HSC), N-cadherin-positive spindle-shaped osteoblasts (SNO cells)

## Abstract

Bone marrow is complex structure containing heterogenetic cells, making it difficult to regenerate using artificial scaffolds. In a previous study, we succeeded in developing honeycomb tricalcium phosphate (TCP), which is a cylindrical scaffold with a honeycomb arrangement of straight pores, and we demonstrated that TCP with 300 and 500 μm pore diameters (300TCP and 500TCP) induced bone marrow structure within the pores. In this study, we examined the optimal scaffold structure for bone marrow with homeostatic bone metabolism using honeycomb TCP. 300TCP and 500TCP were transplanted into rat muscle, and bone marrow formation was histologically assessed. Immunohistochemistry for CD45, CD34, Runt-related transcription factor 2 (Runx2), c-kit single staining, Runx2/N-cadherin, and c-kit/Tie-2 double staining was performed. The area of bone marrow structure, which includes CD45(+) round-shaped hematopoietic cells and CD34(+) sinusoidal vessels, was larger in 300TCP than in 500TCP. Additionally, Runx2(+) osteoblasts and c-kit(+) hematopoietic stem cells were observed on the surface of bone tissue formed within TCP. Among Runx2(+) osteoblasts, spindle-shaped N-cadherin(+) cells existed in association with c-kit(+)Tie-2(+) hematopoietic stem cells on the bone tissue formed within TCP, which formed a hematopoietic stem cell niche similar to as in vivo. Therefore, honeycomb TCP with 300 μm pore diameters may be an artificial scaffold with an optimal geometric structure as a scaffold for bone marrow formation.

## 1. Introduction

In recent years, regenerative medicine developments have made it possible to regenerate bone tissue defects lost due to trauma, tumor resection, infections, and surgery. In bone tissue regenerative medicine, the three main design components in tissue engineering are cells for osteogenesis, their extracellular matrix (scaffolds) for osteoconduction, and a signaling system for osteoinduction, which can be used individually or in combination [1,2]. Among these factors, pluripotent stem cells such as mesenchymal stem cells and iPS cells are used for cell source and some important signaling pathways including Wnt/β-catenin, notch, bone morphogenetic protein-2 (BMP)/transforming growth factor-β (TGF-β), PI3K/Akt/mTOR, and mitogen-activated protein kinase (MAPK) pathways in bone regeneration [3,4,5,6]. The large variety of biomaterials that have been used for in vivo bone tissue engineering have already been reported by others [7,8]. Especially, hydroxyapaptite (HA) and tricalcium phosphate (TCP) are widely applied clinically in bone tissue regeneration from the viewpoint of biocompatibility. They have a composition similar to that of natural bone, good biocompatibility, and osteoconductivity, and can osteointegrate [9]. TCP exists in two major distinct phases of crystals (alpha and beta) similar in their chemical composition but differing in their crystallographic features that confer them different resorption features [10]. While HA has a relatively high crystallinity and is difficult to degrade in vivo, TCP is more degradable than HA and becomes soluble more rapidly [11]. 

Some researchers focus on efforts in chemistry and/or surface properties of scaffolds for the enhancement of bone regeneration and/or osteointegration in bone defects [12]. In addition, many studies have focused on the geometric structure of scaffolds, as it is important not only for the materials of artificial scaffolds but also for the optimal space provided for the differentiation of cells and tissues [13,14]. We have also focused on the importance of the extracellular microenvironment during induction of hard tissue cell differentiation to develop artificial scaffolds that provide an optimal environment and have succeeded in developing a new honeycomb TCP with straight through-pores [15]. In our previous studies, we succeeded in inducing efficient hard-tissue formation by altering the geometry of the linear through pores, and we also succeeded in inducing cartilage and bone tissue formation specifically [16,17]. In this study, we investigated histologically how differences in the pore diameters (75, 300, 500, and 1600 μm) of a honeycomb TCP structure with various final contained amounts of BMP-2 (0, 125, 250, 500, and 1000 ng) influenced hard tissue regeneration. This study’s results showed that bone tissue formation was found in the honeycomb TCP with 300 and 500 μm pore size and a high contained amount of BMP-2 (1000 ng) and that cartilage tissue formation was found in the honeycomb TCP with 75 μm pore size and low contained amount of BMP-2 (125 ng). Therefore, it was suggested that it may be possible to selectively and efficiency induce hard tissue formation, especially bone and cartilage tissue, by altering the geometrical structure of the artificial scaffold (honeycomb TCP). In addition, in this experiment, the honeycomb TCP with a 300 and 500 μm pore size and a high contained amount of BMP-2 (1000 ng) induced bone marrow formation partially [16,17]. 

Thus, although bone and cartilage regeneration research has been conducted using an artificial scaffold, bone marrow regeneration research has not yet been fully developed using artificial scaffolds. The bone marrow contains various heterogeneous cell populations, including hematopoietic and mesenchymal stem cells, that form a homeostatic niche for the maintenance of the bone microenvironment. The bone marrow plays an important role as the major hematopoietic site in mammals. Myelodysplastic syndromes are still intractable hematologic diseases that require allogeneic stem cell transplantation for cure, with fewer clinically available molecularly targeted drugs than other vascular tumors (chronic leukemia, multiple myeloma, malignant lymphoma, etc.). Thus, although myelopoiesis is strongly desired, specific means for regeneration have not yet been clearly defined. Numerous approaches have been made toward the development of an ideal bone marrow scaffold for blood cell production for transfusions and tissue repair as well as for drug testing ex vivo with tissue engineering solutions being one of the greatest emerging options to address these needs [18,19]. The main bone marrow scaffolding is represented by the components of the extracellular matrix (ECM), which provide, depending on their composition, localization, and stiffness, the ideal microenvironment to support hematopoietic stem cell (HSC) differentiation into committed lineages and the release of mature cells into blood [20].

In this study, we reproduced the bone marrow extracellular microenvironment by using honeycomb TCPs with holes of various sizes to search for an appropriate environment for bone marrow formation by honeycomb TCPs and to examine immunohistochemically whether the formed bone marrow can function as a hematopoietic stem cell or mesenchymal stem cell niche. The final goal of this research is to examine the optimal structure of honeycomb TCP scaffold for bone marrow regeneration.

## 2. Materials and Methods

### 2.1. Preparation of Honeycomb TCP Containing BMP-2

**Figure 1:**
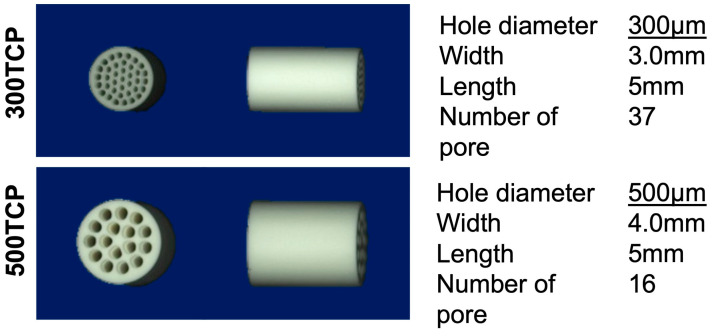
The honeycomb TCP structures used in these experiments. The upper row shows 300TCP and the lower row shows 500TCP.

A 1:2 mixture of CaHPO_4_·2H_2_O powder and CaCO_3_ was prepared, and a slurry was formed by mixing and grinding with water for 24 h in a ball mill. After the slurry was dried, it was pressed in a cylindrical mold with a depth of 5 mm containing through-and-through pores with diameters of 300 μm (300TCP) and 500 μm (500TCP). Each TCP was calcinated by heating to 1200 °C at a rate of 50 °C h^−1^ and holding at that temperature for 1 h. (Figure 1). The details of the honeycomb TCP manufacturing method and properties such as surface properties and crystalline elementary properties were described previously [15].

Each TCP structure was sterilized by autoclave and loaded with BMP-2 diluted to a final contained amount of 1000 ng in Matrigel^®^ (BD Bioscience). Next, these TCPs were implanted into rat femoral muscles [16,17].

### 2.2. Animals and Implantation Procedure

A total of 14 4-week-old healthy male Wister rats were used in this experiment. 300TCP and 500TCP were transplanted into rat femoral muscles. To investigate the optimal structure of honeycomb TCP for bone marrow formation, the animals were randomly divided into 2 groups: 300TCP loaded with BMP-2 diluted to a final contained amount of 1000 ng in Matrigel^®^ and 500TCP loaded with BMP-2 diluted to a final contained amount of 1000 ng in Matrigel^®^.

All experiments were performed in accordance with Okayama University’s Policy on the Care and Use of the Laboratory Animals and approved by the Animal Care and Use Committee. All surgical procedures were performed under general anesthesia in a pain-free state.

### 2.3. Histological Procedure

For histological observations, the implanted TCPs were removed after 3 weeks and fixed in neutral buffered formalin. Next, the specimens were decalcified in 10% ethylenediaminetetraacetic acid for 3 weeks and then embedded in paraffin. Finally, sections were stained with hematoxylin-eosin (HE) and observed histologically.

### 2.4. Bone Marrow Formation Evaluation by Area Measurement

For quantification, the bone marrow formation was measured in five areas chosen from randomly selected regions in HE-stained specimens (200× magnification, *n* = 4) using Image J software (NIH, Bethesda, MD, USA). In this experiment, the area of bone marrow formation was defined as the region containing monocytic cells and vascular structures surrounded by newly formed bone tissue in the TCP pores. In each field, we measured the total area of bone marrow formation in TCP pores and the area of TCP pores, and we calculated the ratio of area of bone marrow area in TCP pores to determine the average of the 5 fields. The obtained average value was compared in each group, and the rate of bone marrow formation was compared for different pore sizes. 

### 2.5. Immunohistochemical (IHC) Staining

IHC staining for CD45, CD34, runt-related transcription factor 2 (Runx2), and c-kit was carried out as follows. The sections were deparaffinized in a series of xylene for 15 min and rehydrated in graded ethanol solutions. Endogenous peroxidase activity was blocked by incubating the sections in 0.3% H_2_O_2_ in methanol for 30 min. Antigen retrieval was achieved by heating in 0.01 mol/L citrate buffer for 1 min. Sections were incubated with primary antibodies as follows: rabbit anti-CD45 (1:100; Abcam, MA, USA), rabbit anti-CD34 (1:500; Abcam, MA, USA), rabbit-anti-Runx2 (1:1000; Abcam, MA, USA), and rabbit anti-c-kit (1:100; DAKO, Santa Clara, CA, USA) overnight at 4 °C. Tagging of primary antibody was achieved by the subsequent application of antirabbit IgG (ABC kit; Vector Laboratories, Inc., Newark, CA, USA). Immunoreactivity was visualized using diaminobenzidine (DAB)/H_2_O_2_ solution (Histofine DAB substrate; Nichirei Biosciences, Inc., Tokyo, Japan), and slides were counterstained with Mayer’s hematoxylin.

### 2.6. Double-Fluorescent IHC Staining

Double-fluorescent IHC staining for Runx2 and N-cadherin, and c-kit and Tie-2 were performed using a primary antibody to anti-N-cadherin (mouse IgG, 1:200; Santa Cruz), and anti-Tie-2 (mouse IgG, 1:100; Abcam). The secondary antibodies used are Alexa Flour 488 antimouse IgG (Abcam, MA, USA) and Alexa Flour 568 antirabbit IgG (Abcam, MA, USA). Antibodies were diluted in Can Get Signal^®^ (TOYOBO Co., LTD., Osaka, Japan). After antigen retrieval, sections were treated with Block Ace^®^ (DS Pharma Biomedical, Osaka, Japan) for 30 min at room temperature. Specimens were incubated with primary antibodies at 4 °C overnight. Then, specimens were incubated with secondary antibody (1:200) for 1 h at room temperature. After the reaction, specimens were stained with 1 μg/mL of DAPI (Dojindo Laboratories, Kumamoto, Japan).

### 2.7. Statistical Analysis

Statistical analysis was performed using one-way analysis of variance and Fisher’s exact tests. A *p*-value < 0.05 was considered statistically significant. All calculations were performed using PASW Statistics 18 (SPSS Inc., Chicago, IL, USA).

## 3. Results

### 3.1. Comparison of Bone Marrow Induction Ability of 300TCP and 500TCP

**Figure 2:**
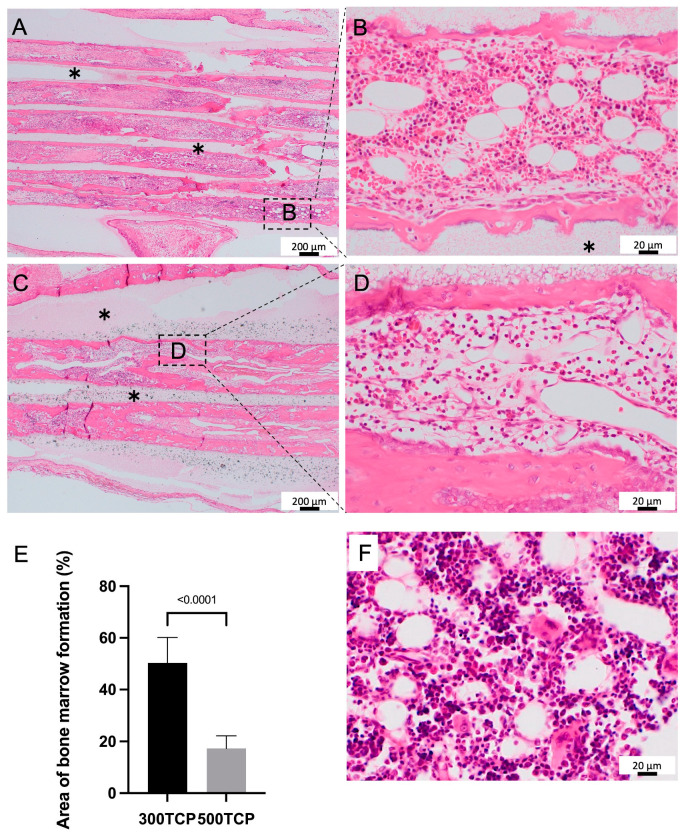
Histological images of formed bone marrow in TCP. (**A**) Low-magnification image of 300TCP. (**B**) Higher-magnification image of corresponding outlined area in (**A**). In the formation tissue surrounded by new bone tissue, the hematopoietic cells, and erythrocytes, sinusoidal vessels were observed in TCP pores, and spindle-shaped cells on the surface of the bone tissue were present. (**C**) Low-magnification image of 500TCP. (**D**) Higher-magnification image of corresponding outlined area in (**C**). The 500TCP showed vigorous laminar bone tissue formation within the pores, which was cortical bone-like in morphology, with bone marrow-like structures. (**E**) Quantification of the bone marrow formation area in the TCP pores. Data are presented as mean ± SD (*n* = 4). (**F**) Histological image of rat normal femoral bone marrow. ∗: TCP.

Bone tissue formation was observed within the 300TCP pores, which was added to the TCP wall. In the formation tissue surrounded by new bone tissue, bone marrow-like tissue with high cell density was observed in 300TCP pores (Figure 2A). The hematopoietic cells lacking cytoplasm and erythrocytes infiltrated into TCP pores, and sinusoidal vessels were observed in TCP pores. Additionally, spindle-shaped cells on the surface of the bone tissue were present, showing a bone marrow-like structure similar to that seen in vivo (Figure 2B). Compared to 300TCP histology, the 500TCP showed vigorous laminar bone tissue formation within the pores, which was cortical bone-like in morphology, with bone marrow-like structures (Figure 2C,D). The area of bone marrow-like structures was larger in the 300 TCP than in the 500 TCP, and in bone marrow tissue in 300TCP, single cells were observed more densely than in 500TCP (Figure 2E). The bone marrow tissue formed in the 300 TCP pore was similar in structure to normal rat femoral marrow (Figure 2F).

### 3.2. Immunohistochemistry Analysis of Formed Bone Marrow Induced by 300TCP

**Figure 3:**
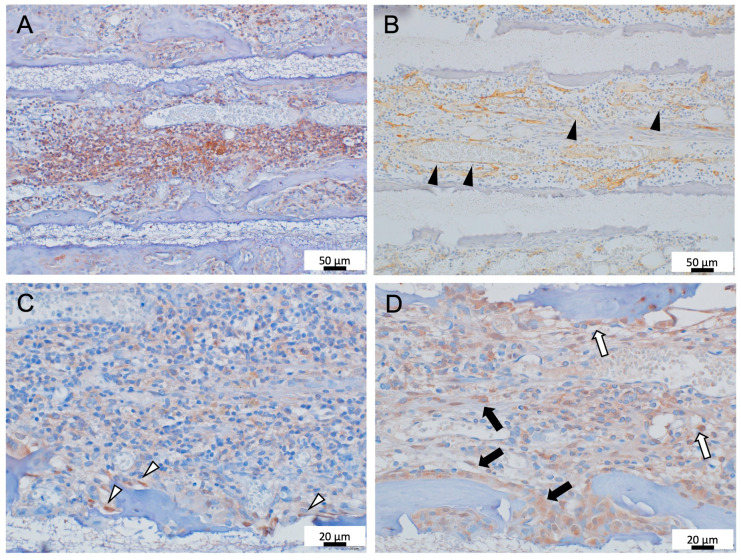
IHC images of 300TCP. (**A**) CD45 immunostaining. CD45-positive hematopoietic cells infiltrated into TCP pores. (**B**) CD34 immunostaining. The abundant vasculature-like structures that were CD34-positive, similar to the sinusoidal vessels, were observed in TCP pores (black arrow heads). (**C**) Runx2 immunostaining. Runx2-positive cells with an oval or spindle-shaped morphology were observed in the surface layer of bone tissue formed (white arrow heads). (**D**) c-kit immunostaining. Oval c-kit positive cells were found within the hematopoietic cells within the TCP pores (white arrows), and clusters of round-to-spindle-shaped c-kit positive cells were observed in the superficial layers of bone tissue formed (black arrows).

Most of the mononuclear cells lacking cytoplasm were CD45-positive, suggesting that they were hematopoietic cells (Figure 3A). There were also abundant vasculature-like structures that were CD34-positive, suggesting a bone marrow structure similar to that found in vivo (Figure 3B) [21].

Bone marrow tissue is deeply involved in bone tissue remodeling [22]. Thus, in this study, the localization of osteoblasts within TCP holes was investigated, and Runx2 was used as a marker for osteoblasts. Runx2-positive cells with an oval- or spindle-shaped morphology were observed in the surface layer of bone tissue formed to add to the TCP wall (Figure 3C).

The hematopoietic stem cells (HSCs) are thought to reside in the bone marrow microenvironment (niche), and c-kit expression on the HSCs is essential for survival of HSCs and maintenance of hematopoiesis [23]. Therefore, we investigated whether an HSC niche exists within the bone marrow-like tissue formed within the TCP. Oval c-kit-positive cells were found within the hematopoietic cells in the bone marrow-like structures within the TCP pores, and clusters of round-to-spindle-shaped c-kit positive cells were observed in the superficial layers of bone tissue formed on the TCP surface (Figure 3D). 

### 3.3. Evaluation of the 300TCP Function as Hematopoietic Stem Cell Niche

#### 3.3.1. Double-Fluorescent IHC for Runx2-N-Cadherin

**Figure 4:**
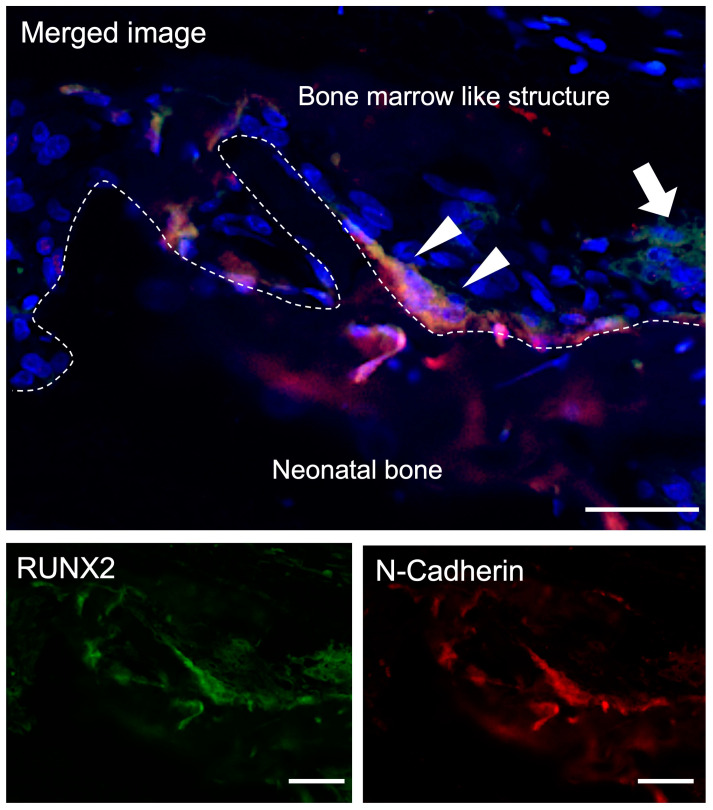
Double-Fluorescent IHC for Runx2-N-cadherin. Osteoblasts in the surface of neonatal bone were Runx2-positive (arrow) and spindle-shaped cells Runx2-N-cadherin double positive (arrowheads) were observed. Blue: DAPI. Green: Runx2, Red: N-cadherin. Scale bars: 20 μm.

It has been reported that osteoblasts within the bone marrow are not uniform, and that N-cadherin-positive spindle-shaped osteoblasts (SNO cells) in the superficial layers of the epiphyseal bone trabeculae are N-cadherin-mediated with hematopoietic stem cells that have the capacity for long-term bone marrow remodeling. Therefore, we investigated the localization of SNO cells within TCP pores [24]. Runx2-positive osteoblasts showing a spindle shape or round shape were observed on the surface of the bone tissue formed within the TCP pores. These Runx2-positive osteoblasts were partially N-cadherin positive (Figure 4).

#### 3.3.2. Double-Fluorescent IHC for c-kit-Tie-2

**Figure 5:**
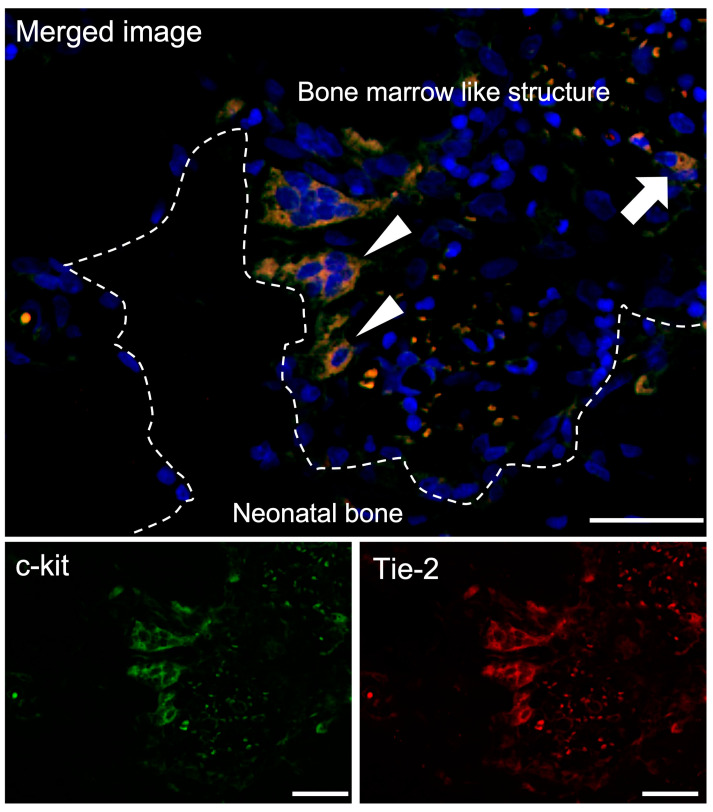
Double-Fluorescent IHC for c-kit-Tie-2. c-kit-Tie-2 double positive cells showing very few small, round-shaped cells were observed in the bone marrow-like structures (arrow), and several clusters of slightly larger cells with similar round morphology that were positive for both c-kit and Tie-2 were observed on the surface of the bone tissue formed in the TCP walls (arrowheads). Blue: DAPI. Green: c-kit. Red: Tie-2. Scale bars: 20 μm.

It has been reported that Tie-2-expressing HSCs are quiescent and antiapoptotic and constitute a cell population that adheres to osteoblasts in the bone marrow niche [25]. c-kit- and Tie-2-positive cells showing very few small, round-shaped cells were observed in the bone marrow-like structures. In addition, several clusters of slightly larger cells with similar round morphology that were positive for both c-kit and Tie-2 were observed on the surface of the bone tissue formed in the TCP walls (Figure 5).

## 4. Discussion

To the best of our knowledge, the present study is the first attempt to regenerate the bone marrow structure where HSCs support the physiologic homeostasis of all blood cells using an artificial scaffold; honeycomb TCP [26,27,28]. The results of this study suggested that it was possible to induce bone marrow structures similar to those in vivo by controlling the geometric structure of engineered biomaterials. In this paper, we propose an optimal geometrical structure to mimic the microarchitecture of bone marrow.

In this study, more vigorous bone marrow formation in TCP pores was observed in 300TCP than in 500TCP (Figure 2). It has been reported that bone tissue formation requires high concentrations of oxygen, while hypoxic conditions are optimal for cartilage tissue formation. Despite the fact that bone marrow is a highly vascularized tissue, pO_2_ in bone marrow is reported to be as low as 32 mmHg or less, with hypoxia at its lowest point of 9.9 mmHg [29]. In this way, it has been suggested that hypoxia condition is advantageous in bone marrow formation. In our previous study, we compared the area and of blood vessels infiltrating into TCP using 75TCP, 300TCP, and 500TCP. This study’s results suggested that the number and area of blood vessels infiltrating into TCP were the lowest and smallest in 75TCP, which induced cartilage formation in TCP. On the other hand, the study results showed that the number and area of blood vessel invasion were the highest in 500TCP [17]. Thus, these results suggested that in the 300TCP, compared to the 500TCP, less oxygen was carried by the blood vessels, resulting in a lower oxygen concentration in the TCP pores and reproducing optimal conditions for myelination. It was thought to mimic a hypoxic environment similar to that within the jawbone. Thus, 300TCP was considered to be the optimal geometry for the bone marrow formation.

In this study, Runx2-positive osteoblasts showing a spindle shape were observed on the surface of the bone tissue formed within the TCP, and these Runx2-positive osteoblasts were also N-cadherin positive (Figure 4). In addition, several clusters of slightly larger cells with a similar round morphology that were positive for both c-kit and Tie-2 were observed on the surface of the bone tissue formed in the TCP walls (Figure 5). Hematopoietic stem cells are maintained in the bone marrow by a special microenvironment called the niche. Niches are broadly classified into the endosteal niche [24], which is composed of specialized osteoblasts, and the vascular niche [30], which is composed of vascular endothelial cells. Osteoblasts were the first cells reported to constitute the hematopoietic niche in mammalian bone marrow; in 1994, undifferentiated blood cells were maintained by coculture with adherent cells of neonatal cranial origin, indicating that the osteoblast derived from neonatal cranial may support hematopoiesis [31]. In 2003, Li et al. reported that osteoblasts function as a niche cell for HSCs in living bone marrow, that some SNO cells and adhere to N-cadherin-positive HSCs on the surface of bone trabeculae, and that stem cell dynamics are regulated by cytokines, adhesion molecules, and extracellular matrices produced by the osteoblasts [24]. Our results showed that c-kit/Tie-2-positive cells were in close proximity to Runx2/N-cadherin-positive cells in bone marrow tissue formed within honeycomb TCP, which is consistent with the in vivo experiments in which Li et al. reported that the BMP signaling pathway via BMPRIA is important for induction of the hematopoietic stem cell niche and that SNO cells on the bone surface are an important component supporting HSCs. In our present experiments, the results of our study were considered to be similar to those of Li et al. in that we used honeycomb TCP and BMP-2. Thus, in the surface layer of bone tissue formed in the TCP pores, HSCs were observed anchoring to SNO cells via N-cadherin, as observed in normal bone marrow, confirming the regulation of HSCs by osteoblasts. 

On the other hand, Keil et al. reported that the majority of BrdU-retaining cells observed histologically by Li et al.’s method, in which approximately 47% of the CD150(+)CD41(-)CD48(-) cell fraction were HSCs, and these cells were barely observable on the bone surface in immunostaining [32], and they reported that vascular endothelial cells constitute a niche for HSCs, as approximately 60% of CD150(+)CD41(-)CD48(-) HSCs adhered to sinusoidal capillaries in the bone marrow cavity (vascular niche) [30]. However, at present, molecules that are essential for HSC maintenance and specifically expressed on vascular endothelial cells have not been identified, and the molecular basis for the HSC niche as an HSC niche is not clear.

In addition, Nagasawa et al. also pointed out that CXCL12-abundant reticular cells (CAR cells) may be a niche for hematopoietic stem cells and immunocompetent cells, since the majority of HSC fractionated cells, early B lymphocyte progenitors, plasma cells, pDCs, and NK cells are attached to CAR cell processes [33,34].

In this study’s results, honeycomb TCP induced endosteal niches; however, it did not induce vascular endothelial cell niches because only a few c-kit-positive cells were present around sinusoidal capillaries in the honeycomb TCP pores, which is clearly less than the number of c-kit-positive cells on the bone surface. The endosteal niche contributes to the long-term maintenance of hematopoietic stem cells by keeping them quiescent and undifferentiated, whereas the vascular niche is assumed to regulate dynamic processes such as proliferation, differentiation, and peripheral mobilization in addition to stem cell maintenance [35]. For example, vascular endothelial cells in the bone marrow support the proliferation and differentiation of hematopoietic progenitor cells, especially megakaryocyte progenitor cells, and injury to bone marrow vessels results in abnormal platelet production [36]. This result could be attributed to the dynamic nature of HSCs around sinusoidal capillaries and the quiescent nature of HSCs on the bone surface, suggesting that the vascular endothelial cell niche may not be completely detectable by immunohistological analysis. In addition, honeycomb TCP did not induce CAR cell niche. This result may be due to the fact that CAR cells have been suggested to be osteoblast/adipocyte progenitor cells, which may have differentiated into osteoblasts under conditions of high BMP-2 concentrations, as in the present experiment. We suggested that the CAR cell niche may be less likely to exist in environments with high concentrations of BMP-2, as in this experiment. Regarding the limitation to this study, we should examine whether honeycomb TCP can function as organs that serve as a long-term and physiologic HSC niche. 

## 5. Conclusions

Honeycomb TCP with 300 μm pore diameters may be an artificial scaffold with an optimal geometric structure as a scaffold for bone marrow formation which works as a hematopoietic stem cell niche. This study suggested that the geometry of artificial scaffold strongly influenced myelopoiesis and that myelopoiesis could be achieved by controlling geometry of artificial scaffold. Therefore, 300TCP is a novel tool for regenerative therapy for bone marrow diseases.

## Data Availability

Data available in a publicly accessible repository.
